# Exploring the secretome of *Corynebacterium glutamicum* ATCC 13032

**DOI:** 10.3389/fbioe.2024.1348184

**Published:** 2024-02-13

**Authors:** Suvasini Balasubramanian, Julie Bonne Køhler, Carsten Jers, Peter Ruhdal Jensen, Ivan Mijakovic

**Affiliations:** ^1^ Microbial Biotechnology and Biorefining, National Food Institute, Technical University of Denmark, Kongens Lyngby, Denmark; ^2^ Novo Nordisk Foundation Center for Biosustainability, Technical University of Denmark, Kongens Lyngby, Denmark; ^3^ Systems and Synthetic Biology Division, Department of Biology and Biological Engineering, Chalmers University of Technology, Gothenburg, Sweden

**Keywords:** *Corynebacterium glutamicum*, secretome analysis, recombinant protein production, α-lactalbumin, β-lactoglobulin

## Abstract

The demand for alternative sources of food proteins is increasing due to the limitations and challenges associated with conventional food production. Advances in biotechnology have enabled the production of proteins using microorganisms, thus prompting the exploration of attractive microbial hosts capable of producing functional proteins in high titers. *Corynebacterium glutamicum* is widely used in industry for the production of amino acids and has many advantages as a host organism for recombinant protein production. However, its performance in this area is limited by low yields of target proteins and high levels of native protein secretion. Despite representing a challenge for heterologous protein production, the *C. glutamicum* secretome has not been fully characterized. In this study, state-of-the-art mass spectrometry-based proteomics was used to identify and analyze the proteins secreted by *C. glutamicum*. Both the wild-type strain and a strain that produced and secreted a recombinant β-lactoglobulin protein were analyzed. A total of 427 proteins were identified in the culture supernatants, with 148 predicted to possess a secretion signal peptide. MS-based proteomics on the secretome enabled a comprehensive characterization and quantification (based on abundance) of the secreted proteins through label-free quantification (LFQ). The top 12 most abundant proteins accounted for almost 80% of the secretome. These are uncharacterized proteins of unknown function, resuscitation promoting factors, protein PS1, Porin B, ABC-type transporter protein and hypothetical membrane protein. The data can be leveraged for protein production by, e.g., utilizing the signal peptides of the most abundant proteins to improve secretion of heterologous proteins. In addition, secretory stress can potentially be alleviated by inactivating non-essential secreted proteins. Here we provide targets by identifying the most abundant, secreted proteins of which majority are of unknown function. The data from this study can thus provide valuable insight for researchers looking to improve protein secretion and optimize *C. glutamicum* as a host for secretory protein production.

## 1 Introduction

Microbial fermentations are emerging as efficient platforms for protein production, as they can produce diverse proteins within a short period of time, while using less land, energy, and water than animal or plant-based systems. Various prokaryotic and eukaryotic expression systems for recombinant protein production have been established. However, the current toolbox for engineering expression hosts may not be suitable for all proteins, particularly those that are complex in nature (Liu et al., 2016).

*Corynebacterium glutamicum* is a Gram-positive, non-sporulating, and facultative anaerobe that is widely known as an industrial workhorse for amino acid production (Schultz et al., 2007; Brinkrolf et al., 2010; Becker et al., 2011; Becker et al., 2018). The host has gained a lot of interest in heterologous protein production due to its attractive features: It is a GRAS host (Generally Recognized as Safe) free of endotoxin, possesses minimal protease activity, and can secrete proteins into the media. In Gram-positive bacteria, proteins are translocated across the intracellular membrane using two transport machineries - the Sec translocon (Sec) and twin-arginine translocation (Tat) pathways (Maffei et al., 2017). The Sec pathway is the primary protein exporting system in bacteria, including *C. glutamicum* that exports unfolded or partially folded proteins across the cytoplasmic membrane. Contrarily, the Tat system is specialized in transporting folded proteins across the membrane (Kikuchi et al., 2009; Teramoto et al., 2011). The secretion of recombinant protein into the culture medium facilitates the downstream isolation of target proteins as it circumvents the need for cell disruption and purification from complex protein lysates. The extracellular medium also provides an oxidative environment supporting disulfide bond formation and hence proper protein folding (Lee et al., 2018; Makrides, 1996).

Different strategies have been implemented to evolve *C. glutamicum* into a robust cell factory for production of amino acids and proteins, including the fine-tuning of relevant metabolic pathways, the development of a cell chassis and of different genetic tools such as optimized expression vectors and promoters for effective target protein production (Freudl, 2017; Lee et al., 2018). Lately, efforts have been put into improving host potential with the use of new secretory pathways, and high-throughput screening technologies (Date et al., 2006; Watanabe et al., 2009; Yim et al., 2016; Balasubramanian et al., 2021). Still, it is not uncommon to fail in producing functional proteins of interest, indicating the need for development of novel strategies to optimize heterologous protein production. To succeed in host optimization, it is critical to have sufficient information about the organism on a systems-wide level including a characterization of its native protein production.

Substantial progress has been made to understand *C. glutamicum* through the establishment of a fully annotated genome (Kalinowski et al., 2003). This has led to a comprehensive understanding of *C. glutamicum* biology, which is critical for the engineering of metabolic pathways and for optimizing genetic elements for improved protein production.

Initial studies on the proteome of *C. glutamicum* ATCC 13032 have been carried out. Hansmeier et al. (2006) established proteome maps of different cellular fractions using 2D gel-based MALDI-TOF and tryptic peptide mass fingerprinting, where 54 proteins were identified in the extracellular fraction. Similarly, Hermann et al. (2001) identified 12 proteins from 40 protein spots on a silver stained 2D gels using matrix assisted laser desorption/ionization-time of flight-mass spectrometry (MALDI-TOF-MS) and electrospray ionization-mass spectrometry (ESI-MS). Only a few of these are well characterized, including *cop1, copB, cmt* (*cmt1*-*cmt6*) from the mycolyl transferase protein family, and the resuscitation-promoting factors (*rpf1* and *rpf2*). Additionally, Yim et al. (2016) identified a major extracellular protein (Cgl1514) during high cell density fed-batch cultivation, secreted using Sec-dependent pathway. The genetic elements of Cgl1514 including the RBS, promoter, and signal peptide are being utilized to facilitate the secretion of heterologous proteins.

Since the initial 2D gel-based secretome studies were performed, the techniques and instrumentation of MS-based proteomics have massively evolved, allowing for more sensitive, quantitative and higher throughput profiling of proteins (Abdallah et al., 2012; Ercan et al., 2023).

**FIGURE 1:**
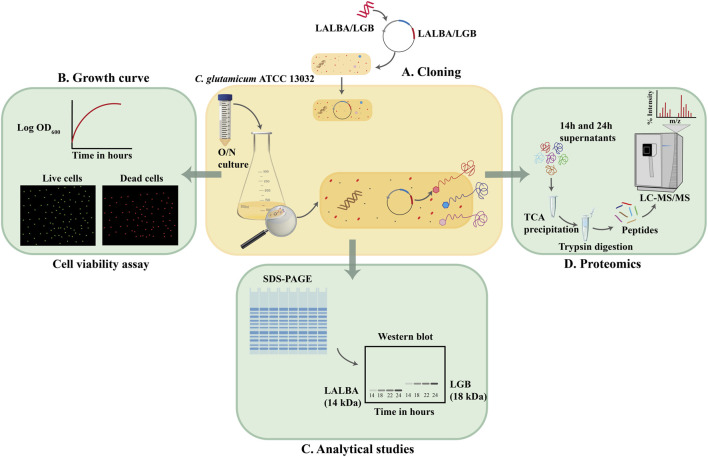
Secretome analysis of *C. glutamicum* ATCC 13032 **(A)**. Genes encoding the whey proteins were inserted in pEC-XC99E vector and used to transform *C. glutamicum* ATCC 13032. **(B)** Growth profiles of the whey protein secreting strains and the wildtype strain without the vector were monitored and cells were assessed for their viability at 2 h intervals. **(C)** Supernatant fractions of the wild type, and whey protein secreting strains were analyzed using SDS-PAGE and Western blot. **(D)** Supernatant fractions of the wild type, and whey protein secreting strains were precipitated and analyzed using LC-MS/MS.

In this study, we used current gel-free LC-MS/MS proteomics methods and intensity-based absolute quantitation (iBAQ) for an in-depth profiling of the *C. glutamicum* ATCC 13032 secretome. The experimental workflow (see Figure 1) involved expression of the β-lactoglobulin gene in *C. glutamicum*. Subsequently, LC-MS/MS analysis was used to identify proteins in the secretome of the wild-type and β-lactoglobulin-expressing strains at different growth phases. Label-free quantification was used to compare the secretome of both strains and at different time points. With this experimental pipeline, we identified a total of 427 proteins in the culture supernatants, of which 148 were predicted to possess a secretion signal peptide. The top 12 most abundant proteins accounted for almost 80% of the secretome.

## 2 Results and discussion

### 2.1 Construction of *C. glutamicum* strains producing whey proteins

**FIGURE 2:**
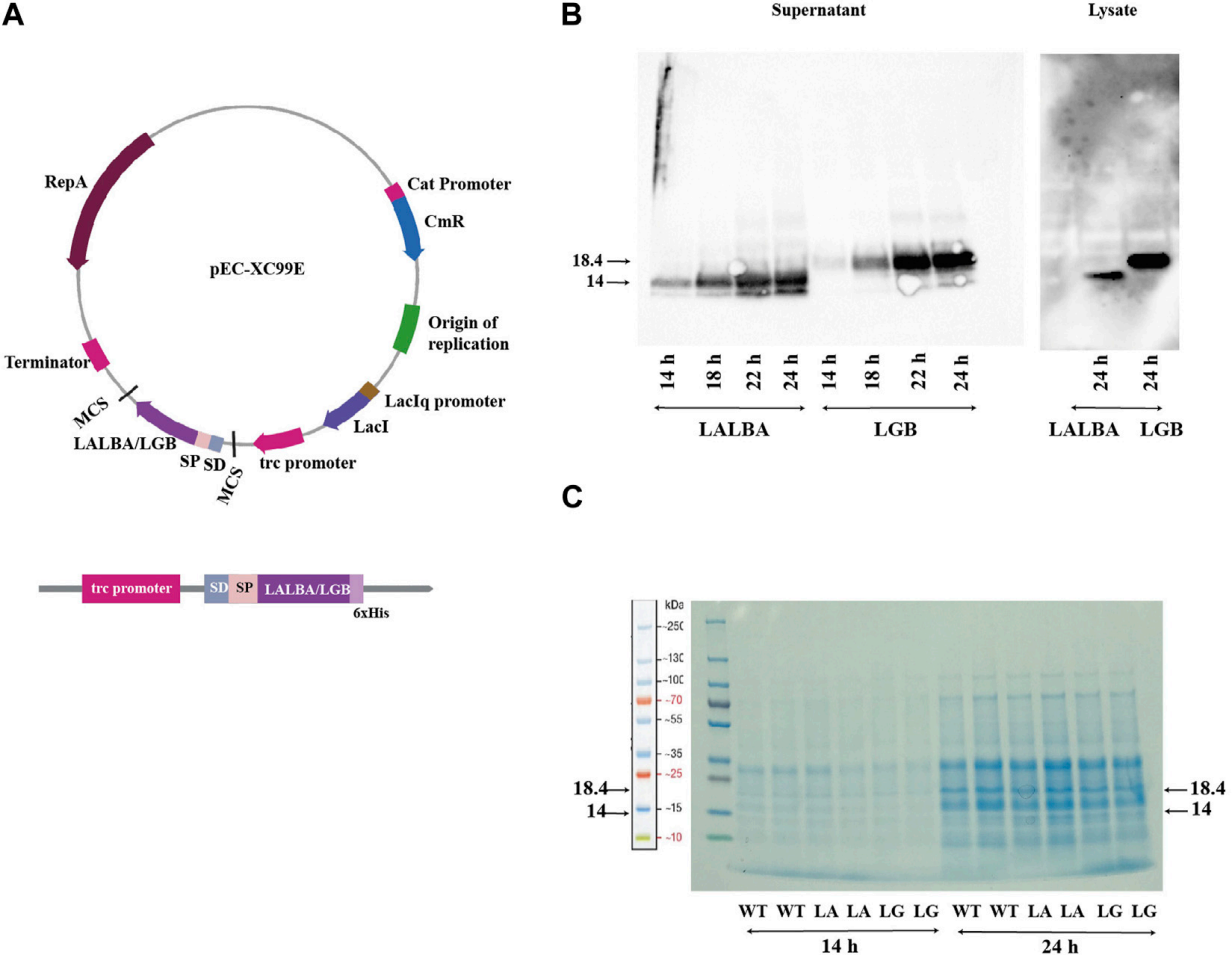
Production of whey proteins: **(A)** The plasmid map of pEC-XC99E with the LALBA or LGB gene inserted. The expression cassette with the translational fusion of signal peptide, LALBA/LGB gene and 6xHis-tag under control of trc promoter and Shine-Dalgarno (SD) sequence is indicated. **(B)** Western blot using anti-His antibody of samples from the α-lactalbumin-producing (LA) and β-lactoglobulin-producing (LG) strains harvested at 14–24 h. **(C)** SDS-polyacrylamide gel of secreted proteins from the wild type (WT), and the α-lactalbumin-producing (LA) and β-lactoglobulin-producing (LG) strains. Samples harvested at 14 and 24 h post-induction are shown.

The shuttle vector pEC-XC99E was used to construct *C. glutamicum* strains secreting the bovine whey proteins α-lactalbumin (LALBA) and β-lactoglobulin (LGB), by placing the LALBA and LGB genes under the control of the IPTG-inducible P_trc_ promoter (as shown in Figure 2A). The CgR0949 signal peptide was fused to the N-terminus of both genes, and a 6xHis tag was fused to their C-terminus. The CgR0949 signal peptide directs transport via the Tat-dependent pathway that can mediate the secretion of properly folded proteins. The secreted proteins were analyzed at every 2 hours during the exponential phase after IPTG induction by precipitating them from the culture supernatants and then subjecting them to SDS-PAGE and Western blotting. In addition, the cells from 24 h cultures were lysed and analyzed. The Western blot analysis demonstrated that the whey proteins were successfully produced and secreted, however there are still residual whey proteins especially β-lactoglobulin in large amounts inside the cell, meaning incomplete translocation by the signal peptide (Figure 2B). Using an anti-His antibody on samples harvested at various time points, we observed bands of approximately 14 kDa and 18.4 kDa in the supernatants, corresponding to α-lactalbumin and β-lactoglobulin, respectively. An increase in target protein concentration was observed over the sampling period from 14 to 24 h post-induction. By comparing samples from the wildtype strain with those of the whey protein producing strains in the SDS-polyacrylamide gel (Figure 2C), it can be concluded that the whey proteins constitute only a small fraction of the total secreted protein.

### 2.2 Growth profile of *C. glutamicum* strains

For the secretome analyses, we chose to continue with the wild type (ATCC 13032) and the β-lactoglobulin-producing strains. Overexpression of heterologous proteins often leads to a metabolic burden that can cause a decreased growth rate of the host organism (Dürrschmid et al., 2008).

**FIGURE 3:**
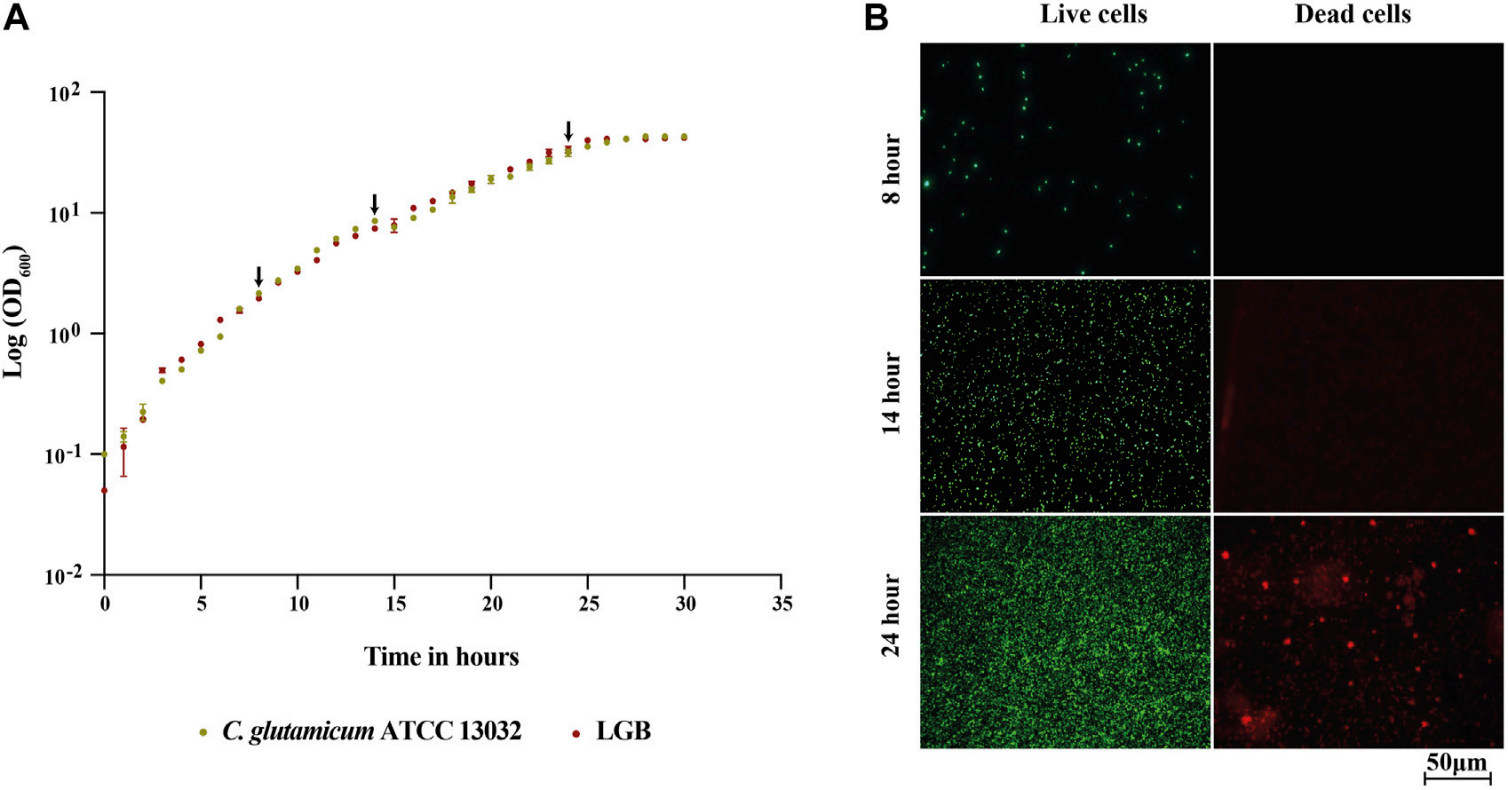
**(A)** Growth profile of the wild type *C. glutamicum* ATCC 13032 strain and its β-lactoglobulin-producing derivative (LGB). **(A)** Growth curve of the wild type (Green) and LGB (Red) strains cultured in CGXII 0.9% BHI medium at 30°C. The averages of three biological replicates are displayed. The arrows indicate the sampling times. **(B)** Fluorescence microscopy of live/dead-stained wild type cells sampled at 8, 14, and 24 h.

The growth profiles of both the strains were found to be similar, and they reached a final OD_600_ of about 40 before entering the stationary phase after approximately 26 h (Figure 3A). Consequently, we concluded that the production of LGB did not seem to affect *C. glutamicum* growth under the tested conditions and thus samples taken at same time points should be comparable between the strains.

Protein in the growth medium can be either a consequence of protein secretion or the release of cytosolic proteins during cell lysis. For the analysis of the secretome, we aimed to minimize the contamination of secreted proteins with the ones from cytosol, which are released due to lysis. To assess the degree of cell lysis and thus potential contamination of secreted proteins in the supernatant, the viability of the cells was tested throughout the fermentation process using a live/dead staining kit, which is based on measuring cellular integrity. The viability of cells during the fermentation process was determined by staining samples taken every 2 h throughout the exponential phase of growth using the BacLight reagent kit. The stained cells were analyzed by fluorescence microscope. This kit includes two types of dye, propidium iodide (PI) and SYTO 9, both of which are used to stain nucleic acids. SYTO 9 produces a green-fluorescent signal and can penetrate all types of cells, enabling the assessment of total cell counts. In contrast, PI produces a red fluorescent signal and can only enter cells with damaged cytoplasmic membranes, which allows for the identification of cells with compromised membranes (Berney et al., 2007). In Figure 3B, the fluorescence microscopy images of samples taken for the WT strain at 8, 14, and 24 h are shown, images from the rest of the time points and for LALBA and LGB are provided in the supplementary file. The binding of SYTO 9 (green) is observed in all pictures confirming presence of live cells, whereas PI (red) is observed only at 24 h but not at 8 and 14 h. The analysis thus indicated that contamination with cytosolic proteins should be minimal at 14 h. The same pattern was observed for the β-lactoglobulin-producing strain (Supplementary Figure S1). Based on this analysis, we chose to analyze the secretome of the strains sampled at 14 and 24 h, with the rationale that 14 h would be primarily secreted proteins and 24 h would include a combination of secreted and cytosolic proteins due to damaged membranes.

### 2.3 Comprehensive analysis of the *C. glutamicum* ATCC 13032 secretome

First, we analysed the genome of *C. glutamicum* containing 3093 protein-coding genes using SignalP to identify signal peptide-containing proteins. Of the 3093 proteins, a total of 205 (6%) were predicted to possess signal peptides (Supplementary Table). Of the 205 proteins, 189 had a Sec-type signal peptide, while 16 had a Tat-type signal peptide.

The composition of the secretome is influenced by a variety of factors, including the growth conditions (temperature, medium, aeration, growth phase at the time of harvest) and expression levels of different genes encoding the secreted proteins (Mattanovich et al., 2009). We were specifically interested in charting the secretome in protein-production conditions and to assess possible differences between the wild type the β-lactoglobulin-producing strains. To assess this, we cultured the strains in protein production conditions and took samples at 14- and 24-hours post-induction corresponding to mid- and late-exponential phase (Figure 3A).

From all experiments combined, peptides corresponding to 539 unique proteins were identified in the supernatant of *C. glutamicum*. For label-free quantification, only proteins that were quantified in both the duplicates, and for which at least two unique peptides were identified, were included. This filtration process resulted in the quantification of 427 proteins, with 148 of these possessing a signal peptide. Hereof, 72 were predicted to be secreted via the Sec pathway, 69 via Sec Lipo pathway and remaining 7 via the Tat pathway (Supplementary excel file). Sec signal peptides are more related to the general protein secretion pathway, while Sec Lipo signal peptides are associated with lipoproteins that are anchored to the bacterial cell membrane. The remaining 279 proteins may be proteins that are secreted using unconventional/uncharacterized pathways, cytosolic proteins due to cell lysis or membrane proteins released, e.g., as a result of proteolytic cleavage.

**FIGURE 4:**
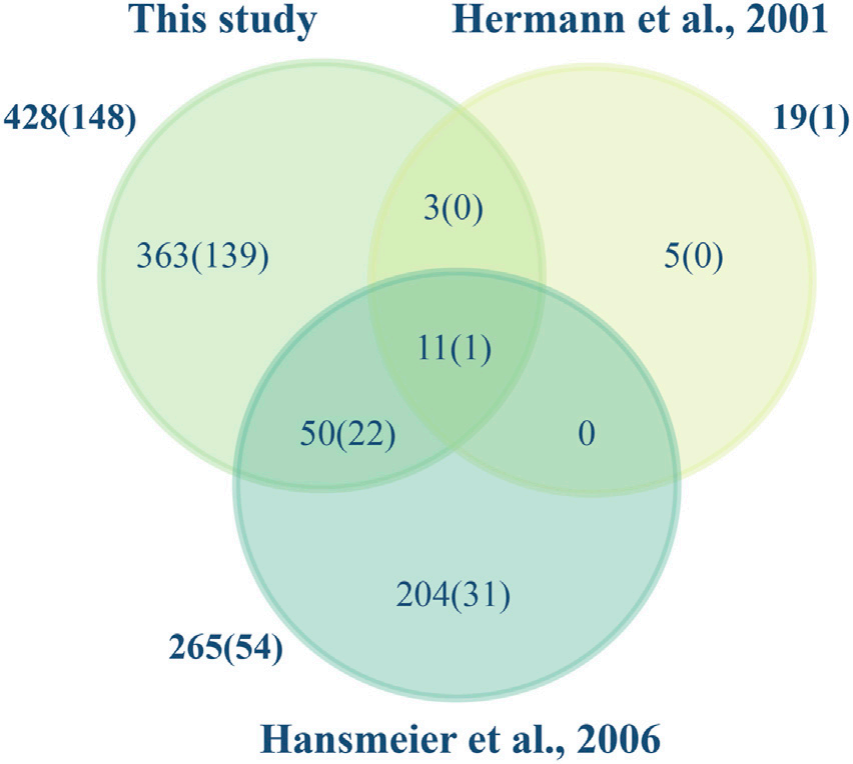
Secreted proteins identified in this and other studies. The Venn diagram illustrates the *C. glutamicum* ATCC 13032 proteins identified in this study and those of Hermann et al., 2001; Hansmeier et al., 2006. Total numbers identified and in parenthesis those containing a signal peptide are shown.

According to UniProt, 98 of the total identified proteins are uncharacterized proteins (UniProt Consortium, 2015). The proteome of *C. glutamicum* ATCC 13032 has been characterized earlier using 2-D gel-based techniques where the cytosolic, cell surface and the extracellular fraction of the host have been mapped. Using this approach, proteins are separated based on their isoelectric point and molecular weight in 2D gels, which can pose challenges in accurately quantifying proteins due to differential protein processing and comigrating spots (Gygi et al., 2000). In the study by Hansmeier et al. (2006), 54 proteins were identified in the extracellular fraction of *C. glutamicum*, of which 28 were also detected in our analysis. Conversely, only one protein out of the six identified by Hermann et al. (2001) was found in our study (Figure 4). Although Hansmeier et al. (2006) reported that all the identified proteins possessed a signal peptide, it is absent in 4 of these proteins (Membrane fusion protein, D-alanyl-D-alanine carboxypeptidase, metalloendopeptidase, DtxR) based on SignalP v.6. These discrepancies are often due to signal peptide predictions by different versions of SignalP. It can be attributed to updates and improvements in the algorithms, refined models, improved accuracy and sensitivity, etc. to distinguish signal peptides from uncleaved signal anchors (Garcion et al., 2021). Conversely, label-free quantification (LFQ) enables determining the relative abundance of proteins amongst different biological samples based on peptide intensity profiling. It provides a more precise and reliable representation of protein abundance by detecting proteins across a wide dynamic range of concentrations without the limitations of variability in spot intensity and protein separation (Magdeldin et al., 2014). Using LFQ, we have identified 78% of the predicted secretome (proteins with a signal peptide) and characterized the abundance of these proteins in the extracellular proteome. We have thus in this study substantially increased the number of experimentally validated proteins secreted by *C*. *glutamicum*.

**FIGURE 5:**
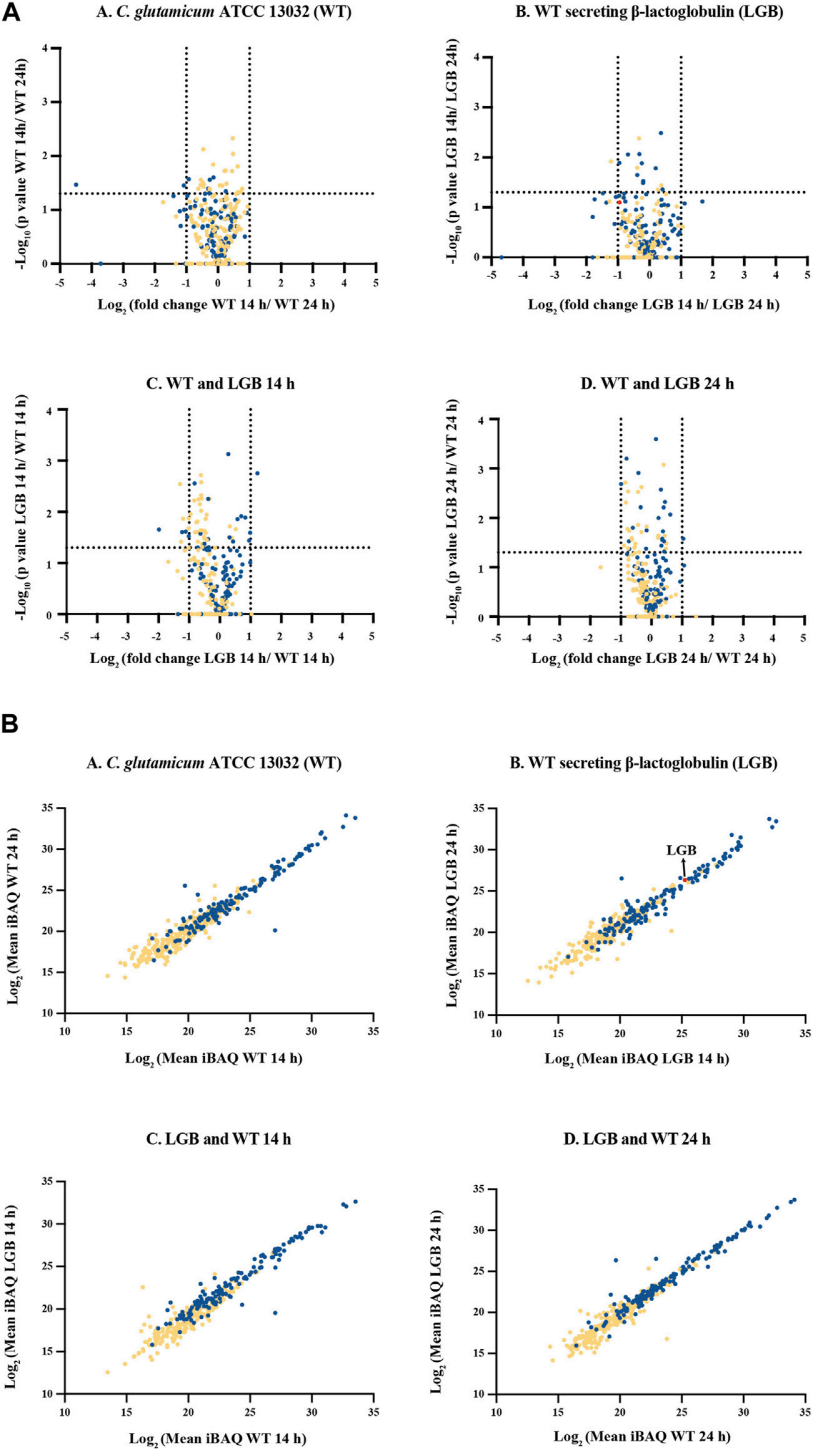
**(A)** Volcano plot of secreted proteins in the secretome of *C. glutamicum* strains WT and LGB at 14 and 24 h. **(A–D)**– Comparison of proteins from within and amongst the WT and LGB strains at 14 and 24 h. The dotted lines correspond to a fold-change cutoff of two-fold (Log_2_ ≤ −1 or ≥1) and a *p*-value of 0.05. **(B)** Protein abundance of secreted proteins. **(A–D)**- Comparison of protein iBAQ values (a proxy for protein abundance) for supernatant at 14 and 24 h for the wildtype and the strain secreting LGB, and between the two wildtype and LGB secreting strains. Each data point represents a protein. The red data point represents β-lactoglobulin. The blue and yellow data points correspond to proteins with and without a signal peptide, respectively.

This label-free quantification approach enabled us to investigate the effects of heterologous protein expression on the host secretome, as well as the differences in secretome composition between mid- and late-exponential phases (Figure 5A).

In general, only very few proteins exhibited a significant difference in response to different growth phases or by expressing heterologous protein. These are cellular machinery proteins since they are involved in various cellular processes, including transport, energy generation, redox reactions, and metabolic pathways.

Figure 5B illustrates a protein abundance comparison in the culture supernatants of the strains. The plots demonstrate a strong correlation in absolute protein concentrations between strains and time-points. Secreted proteins occupy the upper portion of the linear plot due to their higher relative abundance compared to cytoplasmic proteins, likely resulting from cell lysis. Notably, this study has uncovered proteins, previously unreported, that constitute a substantial portion of the extracellular proteome. The top 12 most abundant proteins secreted using Sec-pathway constitute approximately 80%–84% of the total secretome, with Q8NPI1, Q8NP21, and Q8NQ03 being the most abundant, contributing an average of approximately 22%, 20%, and 13% of the total secretome, respectively (Table 1). The list of all the proteins identified and quantified in this study can be found in the supplementary file.

**TABLE 1:** List of most abundant proteins identified in the secretome of *C. glutamicum* ATCC 13032.

Accession no.	Signal peptide	Description	Reported in the literature
Q8NP21	Sec	Hypothetical membrane protein	-
Q8NPI1	Sec	Uncharacterized protein	-
Q8NQ03	Sec	Uncharacterized protein	-
Q6M6W5	Sec	Resuscitation-promoting factor Rpf1	Hansmeier et al. (2006)
Q8NU62	Sec	Uncharacterized protein	-
Q8NRS3	Sec	PorB	-
Q8NPW5	Sec	Uncharacterized protein	-
Q8NQT2	Sec	Uncharacterized protein	Yim et al. (2016)
Q6M6N7	Sec	Resuscitation-promoting factor Rpf2	Hansmeier et al. (2006)
P0C1D6	Sec	Protein PS1	Hermann et al., 2001; Hansmeier et al., 2006
Q8NSV8	Sec	Uncharacterized BCR	-
Q8NMV3	Sec lipo	ABC-type transporter	-

Q8NP21 is a hypothetical membrane protein, while Q8NPI1 and Q8NQ03 are uncharacterized proteins. Followed by these are the resuscitation promoting factor genes (RPF) making up 6%–9% of the abundance in our secretome data. Rpf1 and rpf2, which were initially considered non-essential cell surface genes, have shown to be critical in stimulating growth. The deletion of these genes resulted in a prolonged lag phase after storage (Hartmann et al., 2004). Of the top 12 proteins, the unknown proteins were used as a query in BLASTp against non-redundant and reference protein sequences (Altschul et al., 1990). The resulting hits were all hypothetical and/or uncharacterized proteins. Thus, the function of these proteins remains unknown.

One of the abundant proteins in the *C. glutamicum* secretome dataset, Q8NQT2, was not predicted to have a signal peptide by SignalP. The protein was previously identified as Cgl1514, a highly abundant protein in the extracellular fraction by Yim et al. (2016) (Supplementary Figure S2). Upon examining the sequence, we surmise that the annotated start site is likely wrong. If assuming that translation is initiated 105 bp downstream, the corresponding protein would have a sequence identified as a signal using SignalP (Supplementary Figure S2). Additionally, we noticed that 6 bp upstream of the potential start codon, a putative RBS site exists (AGGAG).

In the work of Yim et al. (2016), Cgl1514 was reported to constitute approximately 51% of the total extracellular protein content of *C. glutamicum* ATCC 13032. However, our study revealed a relatively lower mean abundance of Cgl1514 at 2.6% across all the samples analyzed. Whether this difference is due to, e.g., different production conditions is unclear.

**FIGURE 6:**
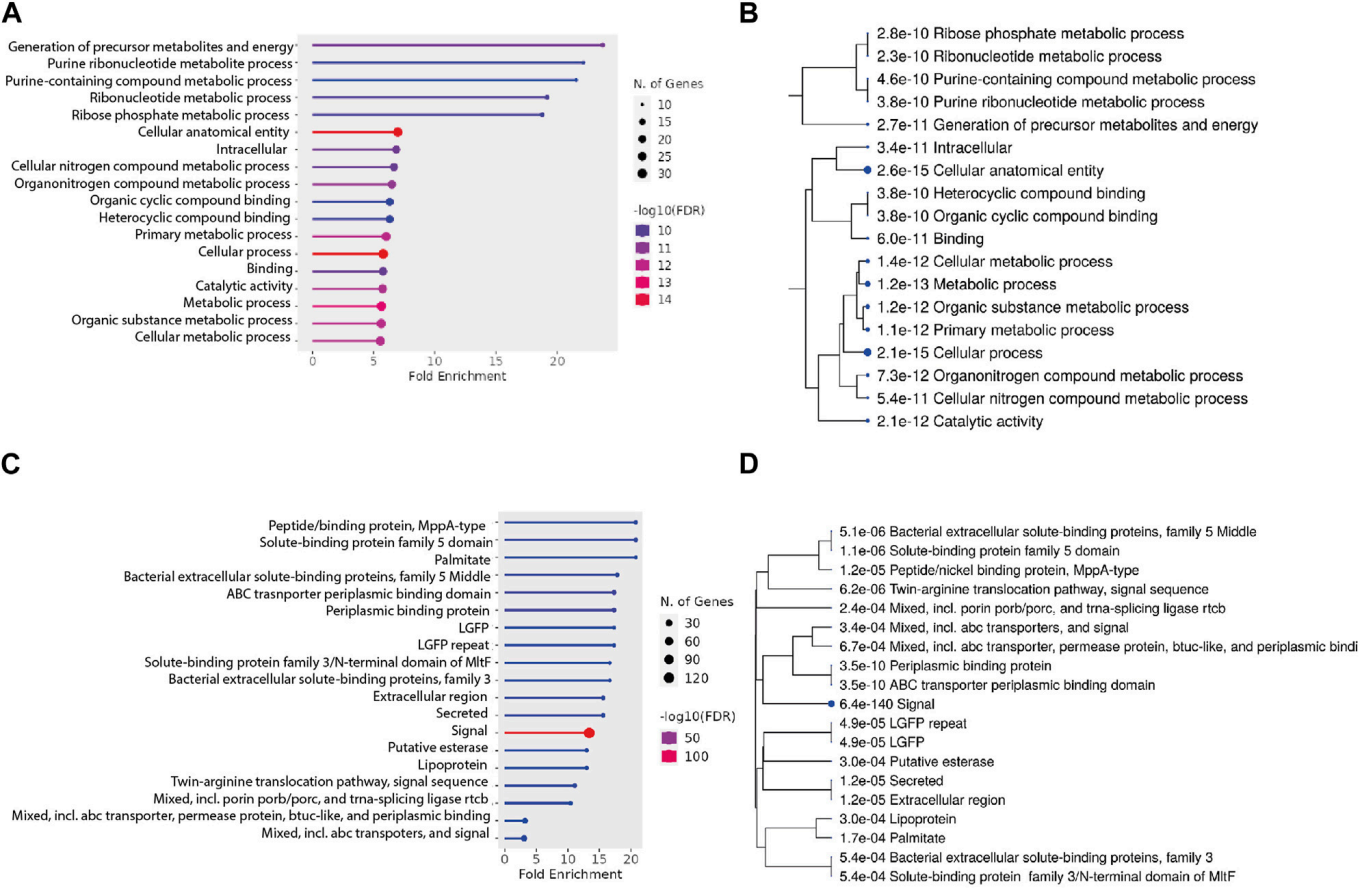
**(A,B)** Pathway analysis for proteins without a signal peptide. **(A)** Fold enrichment analysis of the genes without a secretion signal identified in the secretome. Genes are grouped into their respective pathways. **(B)** A hierarchical clustering tree summarizes the correlation among significant pathways listed in the enrichment figure **(A)**. **(C, D)** Pathway analysis for proteins with a signal peptide. **(C)** Fold enrichment analysis of the genes without a secretion signal identified in the secretome. Genes are grouped into their respective pathways. **(D)** A hierarchical clustering tree summarizes the correlation among significant pathways listed in the enrichment figure **(C)**. Pathways with many shared genes are clustered together. Bigger dots indicate more significant *p*-values.

It was observed that the proteins devoid of signal peptides generally were found in low abundance. While there are mechanisms for protein export that are independent of signal peptides, we considered it is likely that a substantial part of the identified proteins would be cytosolic proteins. To support this further, we evaluated the functional categories of the proteins using ShinyGO Version 0.77 (Ge et al., 2020). The top enriched pathways are potential biological processes related to cellular functions and metabolism as shown in Figures 6A, B. Genes enriched into the pathways include—purine containing compound metabolic processes and ribonucleotide metabolic processes. Other enriched pathways include cellular anatomical entity, intracellular components, cellular nitrogen compound metabolic process, and more. These pathways are related to cellular processes and metabolic activities that occur within the cells. The identification of proteins from these pathways thus implies that the observed protein might be found in the secretome due to a low level of cell lysis.

Similarly, the enrichment analysis of proteins with signal peptides (Figures 6C, D) revealed identification of several significant pathways based on their FDR values, gene counts, and fold enrichments. Notable findings include the presence of proteins related to peptide/nickel binding (MppA-type), solute-binding protein family 5 domain, palmitate binding, and bacterial extracellular solute-binding proteins. Additionally, pathways associated with ABC transporter periplasmic binding, lipoproteins, and twin-arginine translocation were enriched. These pathways point towards potential cellular processes such as transport, binding, and signal sequences. The diverse range of identified pathways underscores the complexity of the cellular mechanisms at play in our study.

We had expected that heterologous protein production would alter the secretome composition, but we did not observe that. The relative abundance of the target protein, β-lactoglobulin, was very low, constituting only 0.13% and 0.14% of the total secretome in the 14-hour and 24-hour LGB culture supernatants, respectively. Whether this modest production level was too small to affect the secretome composition is unclear. There could be many reasons for the relatively low production, such as, e.g., key regulatory elements involved in gene/protein expression such as signal peptide, type/nature of the protein, promoter, or a combination of these elements (Doane and Elemento, 2017).

### 2.4 Bottlenecks and opportunities for improving protein production using *C. glutamicum* secretome

*Corynebacterium glutamicum* is a promising host for the production of heterologous proteins, but low protein yields and high levels of native protein secretion remain significant bottleneck to its performance. The quantitative data from this study, can be leveraged to identify efficient signal peptides and, potentially, strong promoters that can increase the transcription of the gene encoding the target protein, leading to higher mRNA levels. From the secretome data, *C. glutamicum* produced low levels (0.1%) of two types of proteases, trypsin-like serine proteases, and membrane-associated zinc-dependent proteases, with the former lacking a signal peptide. However, no activity has been observed against the substrates skimmed milk, casein, and collagen (Joliff et al., 1992). It cannot, however, be ruled out that these proteases might still impact heterologous protein production levels in *C. glutamicum*. Consequently, inactivating the proteases could potentially improve target protein production.

The use of metabolic engineering strategies to optimize the production of proteins in *C. glutamicum* can also be complemented with the use of advanced proteomics techniques to better understand the cellular processes and components involved in protein production and secretion, and to develop targeted approaches for improving these processes. Previous studies in *C. glutamicum* have shown the effectiveness of reducing the genome size by deleting specific genes. For example, the prophages CGP1, CGP2, and CGP3 were successfully deleted, resulting in a genome-reduced strain with improved transformation efficiency and which in turn increased protein production due to high copy number (Baumgart et al., 2013). Genome reduction might not directly correlate to improved protein expression; however, it could be attributed by a gene cluster that is being knocked out. This prophage-free strain exhibited an increase similar to the strain ΔcglMRR which lacks the restriction modification system in CGP3 gene. Similar trend has been observed in *B. subtilis* MGB874 strain with a 20% reduced-genome, where a major gene rocDEF-rocR alone led to a significant increase in the protein yield. However, with the deletion of other genes, the specific productivity of the strain also increased (Manabe et al.). One could also attempt to optimize the host based on our secretome data. The cellular resources and energy required for protein secretion may be primarily allocated towards the native proteins, limiting the availability of these resources for the efficient secretion of the target protein.

Our analysis revealed a number of abundant, uncharacterized proteins that do not have any assigned function or involvement in known cellular and molecular pathways. Provided that the proteins are non-essential, their inactivation might free up resource for production and secretion of heterologous target proteins. This could be a promising strategy to develop a more streamlined and efficient cell chassis for improved secretion and yield of heterologous proteins (Nombela et al., 2006; Mattanovich et al., 2009).

MS-based proteomics contributes to a deeper understanding of the cellular processes and optimization of the protein production in various hosts by serving as a powerful and robust tool for protein identification, characterization, and quantification. In this study, we identified, characterized, and quantified a total of 427 distinct proteins, encompassing both extracellular and cytoplasmic proteins present in the supernatant. Among the 205 proteins predicted to be extracellular, approximately 72% were successfully identified and quantified based on their abundance.

The knowledge from this study can facilitate the identification of efficient signal peptides and promoters belonging to the abundant native proteins, as well as the development of genome-reduced strains by knocking out non-essential genes. Our findings provide valuable insights into the *C. glutamicum* secretome and highlight the potential for future studies to further explore the mechanisms and regulation of protein secretion in this organism. Furthermore, the identification of uncharacterized proteins and their potential impact on heterologous protein production suggests a new direction for developing more efficient protein production systems. These findings have significant implications for the biotechnology industry and offer exciting opportunities for the development of novel strategies for improving protein production.

## 3 Materials and methods

### 3.1 Bacterial strains, media, and growth conditions

In this study, *C. glutamicum* wild type ATCC 13032 was engineered to secrete the whey proteins - α-lactalbumin (strain referred to as LALBA) and β-lactoglobulin (strain referred to as LGB). All overnight cultures were grown in brain heart infusion broth (BHI, Sigma, St. Louis, MO, United States).

For protein expression, CGXII medium (Unthan et al., 2014) supplemented with 0.1% BHI was used. The media composition per liter is 1 g BHI, 20 g (NH_4_)_2_SO_4_, 1 g K_2_HPO_4_, 1 g KH_2_PO_4_, 5 g urea, 13.25 mg CaCl_2_⋅2H_2_O, 0.25 g MgSO_4_⋅7H_2_O, 10 mg FeSO_4_⋅7H_2_O, 10 mg MnSO_4_⋅H_2_O, 0.02 mg NiCl_2_⋅6H_2_O, 0.313 mg CuSO_4_⋅5H_2_O, 1 mg ZnSO_4_⋅7H_2_O, 42 g MOPS, 0.2 mg biotin, 30 mg protocatechuic acid and 40 g D-glucose. For the preparation of electro-competent cells, the standard procedure in the Handbook of *C. glutamicum* was followed (Eggeling and Bott, 2005). The antibiotic concentration was 10 μg/mL for chloramphenicol. For induction of protein production, 1 mM Isopropyl ß-D-1-thiogalactopyranoside (IPTG, Sigma-Aldrich, St. Louis, MO, United States) was used. All the strains were grown at 30°C at 200 RPM in 500 mL baffled shake flasks.

### 3.2 Plasmid construction

Gene fragments of α-lactalbumin and β-lactoglobulin (with the UniProt IDs P00711 and P02754 respectively) were synthesized by IDT. The N-terminus of the gene fragments contained SD sequence of the tpiA gene (30 bp before the start codon) from *C. glutamicum,* followed by the TAT type signal peptide from Cgr0949. 6xHis tag was fused to the C-terminus of the genes. The genes were designed to have overlapping regions of the vector pEC-XC99E to clone using Gibson assembly.

For the construction of the strains secreting α-lactalbumin and β-lactoglobulin (LALBA and LGB respectively), pECALA, and pECLGB by introducing the corresponding gene fragments between the BamHI and XbaI sites in pEC-XC99E. The vectors were then used to transform *C. glutamicum* ATCC 13032 wild-type strain.

### 3.3 Growth curve

Strains were inoculated to a start OD_600_ of 0.05 and grown for 48 h, and their optical density (OD_600_) was measured every hour until the cultures reached the stationary phase at around 30 h. Samples were drawn out simultaneously for further analysis. For analyzing the proteins on SDS-PAGE, and western blot, 2 mL of the samples were collected at 8, 14, and 24 h.

### 3.4 Live/dead cell viability assay

Live/dead staining was done according to the instructions on the *Bac*Light bacterial viability kit from Invitrogen (Cat. L13152). Samples were taken every 2 hours during the exponential phase and the cells were stained and analyzed using a fluorescence microscope (Leica DM4000 B).

### 3.5 Protein expression and extraction

For protein expression of WT (no heterologous gene) and whey protein strains, an overnight culture in BHI media supplemented with 1% glucose was used. The subcultures were grown in CGXII media supplemented with BHI, and IPTG in 500 mL baffled shake flasks with a culture volume of 40 mL. The cultures were centrifuged at 5000 x g for 25 min at 4°C, and the supernatant was processed for subsequent use.

The extracellular proteins were extracted from the supernatant as described by (Bittel et al., 2018). To the supernatant, a complete EDTA-free protease inhibitor cocktail was added to prevent proteolysis and centrifuged at 7,000 *g* for 1 h, at 4°C. The supernatants were filtered using 0.2 μm sterivex filters (Merck Millipore). Trichloroacetic (10% w/v) acid was added to the supernatant under constant stirring and incubated overnight at 4°C to precipitate the proteins. The protein pellets were collected after spinning down for 1 h, 5,500 g at 4°C. The precipitated protein pellets were washed with 80% acetone twice and 100% acetone followed by incubation on ice for 5 min and centrifugation at 5,500 *g* for 10 min at 4°C. The pellets were air dried and subsequently solubilized in 400 μL rehydration buffer (8 M urea, 50 mM Tris-base, 20 mM DTT, and 1% sodium deoxycholate). After measuring the final protein concentration, the samples were flash-frozen using liquid nitrogen and stored at −20°C.

### 3.6 Qualitative and quantitative analysis

#### 3.6.1 Bradford assay

Bradford assay (Sigma- Aldrich) was performed following the manufacturer’s instructions to measure the total protein concentration with bovine serum albumin (BSA) as a standard.

#### 3.6.2 SDS-PAGE

The samples were diluted with 4x laemmli buffer and 100 μM DTT (Bio-Rad Laboratories), and boiled at 95°C for 10 min. Proteins were subsequently separated on a gradient (4%–20%) mini-PROTEAN TGX precast polyacrylamide gels (Bio-Rad Laboratories) and analyzed on a Bio-Rad imager.

#### 3.6.3 Western blot

iBlot^TM^ 2 mini transfer stacks containing PVDF membranes were used for blotting using the iBlot^TM^ 2 gel transfer device with preset template run settings P1 (25 V, 6 min) used. The blotted membrane was washed thrice with TBST buffer (Tris buffered saline solution, 0.1% tween20) for 5 min and then transferred to TBST containing 5% BSA for blocking at room temperature for 1 h on a rocking shaker. After blocking, the membranes were transferred to the blocking buffer containing Rabbit Anti-his antibodies (0.1 μg/mL) and incubated overnight at 4°C on a rocker. After incubation with the primary antibody, the membrane was washed thrice with TBST for 5 min and then transferred to the blocking buffer containing goat anti-rabbit secondary antibody (1:5000) and incubated for 1 h. Post incubation, the membrane was washed thrice with TBST and incubated with Supersignal^TM^ West Pico chemiluminescent substrate (Thermofisher). The membrane was visualized using AI600 Imager (GE Healthcare).

### 3.7 Sample preparation for mass spectrometry

In-solution tryptic digest of the samples was prepared using a filter-aided sample preparation method. A total of 100 μg of protein was exchanged into UA buffer (8 M urea, 100 mM Tris, pH - 8.0) to remove DTT and sodium deoxycholate using 30 kDa cut-off Microcon filters (Merck Millipore) for a couple of spins centrifuged at 14000 x g for 15 min. Alkylation was carried out in the filters using UA buffer with 50 mM chloroacetamide and incubated in the dark for 20 min. Post incubation, the filters were washed with UA buffer thrice followed by additional washes with 50 mM ammonium bicarbonate (AB buffer) twice. The proteins were then digested overnight at 37°C with trypsin (Sigma Aldrich) using AB buffer (1:50 enzyme: protein ratio). Peptides were collected by centrifugation and desalted on C18 stage tips.

### 3.8 Mass spectrometric analyses

Desalted samples were injected into Orbitrap Exploris 480 mass spectrometer (Thermo Scientific) using a CapLC system (Thermo scientific). First samples were captured at a flow of 10 μL/min on a pre-column (µ-precolumn C18 PepMap 100, 5 μm, 100 Å) and then at a flow of 1.2 μL/min the peptides were separated on a 15 cm C18 easy spray column (PepMap RSLC C18 2 μm, 100 Å, 150 µmx15 cm). The applied gradient went from 4% acetonitrile in water to 76% over a total of 60 min. While spraying the samples into the mass spectrometer the instrument operated in data-dependent mode using the following settings: MS-level scans were performed with Orbitrap resolution set to 60,000; AGC Target 3.0e6; maximum injection time 50 ms; intensity threshold 5.0e3; dynamic exclusion 25 s. Data-dependent MS2 selection was performed in Top 20 Speed mode with HCD collision energy set to 28% (AGC target 1.0e4, maximum injection time 22 ms, Isolation window 1.2 m/z).

### 3.9 Data analysis

MS raw files were processed with the MaxQuant software (2.0.1.0) using the Andromeda search engine (Cox et al., 2011). MS/MS raw files were searched against the Uniprot *C. glutamicum* ATCC 13032 database (UP000000582) and the LGB protein sequence with the LFQ and iBAQ options enabled. Trypsin was set as the digestion mode with a maximum of two missed cleavages. Oxidation of Met and acetylation of protein N-termini were set as variable modifications, while carbamidomethylation of Cys residues was selected as a fixed modification. Search results were filtered by the target-decoy method with a false discovery rate (FDR) cutoff of 1%.

The MaxQuant ProteinGroup output file was further processed with the Perseus software (1.6.15) for statistical analysis (Tyanova et al., 2016). The MS analysis resulted in the identification of 539 proteins after reversed and potential contaminants were removed. Only proteins that were quantified in both experimental duplicates, and for which at least two unique peptides were identified, were used for quantification analysis. This filtration process resulted in the quantification of 427 proteins. Protein LFQ and iBAQ intensity values of the proteins were log_2_ transformed to compare protein intensity across the samples, and protein abundance within the sample respectively. To compare protein LFQ intensities between different samples, a student two-sample *t*-test was used with a *p*-value threshold of 0.05. Samples from the same strain at different time points were compared with a paired t-tests while unpaired t-tests were employed for comparing between the strains. For the absolute protein quantification within sample, mean iBAQ values for biological duplicates were normalized to the sum of all iBAQ values for a given sample, to generate a relative iBAQ (riBAQ) value for each protein (Shin et al., 2013). The processed data from Perseus (supplementary excel file) was exported to GraphPad prism for generating plots.

### 3.10 Bioinformatic analyses

The proteome of *C. glutamicum* ATCC 13032 (UP000000582) was retrieved from UniProt. The proteome was run on SignalP 6.0 to predict the proteins with signal peptide (Teufel et al., 2022). The proteins with a signal peptide were matched against our raw data and processed in Perseus. NCBI BLAST was used for sequence alignment.

## Data Availability

The datasets presented in this study can be found. The data presented in the study are deposited in the ProteomeXchange repository, accession number PXD047109.

## Supplementary material

SUPPLEMENTARY FIGURE S1 Fluorescence microscopy of live/dead stained C. glutamicum ATCC 13032 (WT), WT secreting α-lactalbumin, and WT secreting β-lactoglobulin cells sampled every two hours in the exponential phase.

SUPPLEMENTARY FIGURE S2 Sequence alignment of Cgl1514 and Q8NQT2 gene. Supplementary excel file lists all the identified proteins and their peptide scores.
