# Supplementary material for: Exploring the secretome of Corynebacterium glutamicum ATCC 13032
Source: Front Bioeng Biotechnol. 2024 Feb 13;12:1348184. doi: 10.3389/fbioe.2024.1348184 (PMC10896948; doi:10.3389/fbioe.2024.1348184)
Supplement: Supplementary file 2 [file DataSheet1.docx]

Supplementary Material

# Supplementary Figures and Tables

## Supplementary Figures

The supplementary figure 1 shows the fluorescent images of all the strains from live/dead staining. The binding of SYTO 9 (green) is observed in all pictures and the intensity of the green-fluorescent signal increases in correlation with the exponential phase of the strains confirming presence of live cells. From the figure, it can also be observed that there are more dead cells present in the WT samples relative to the other strains expressing the whey proteins.


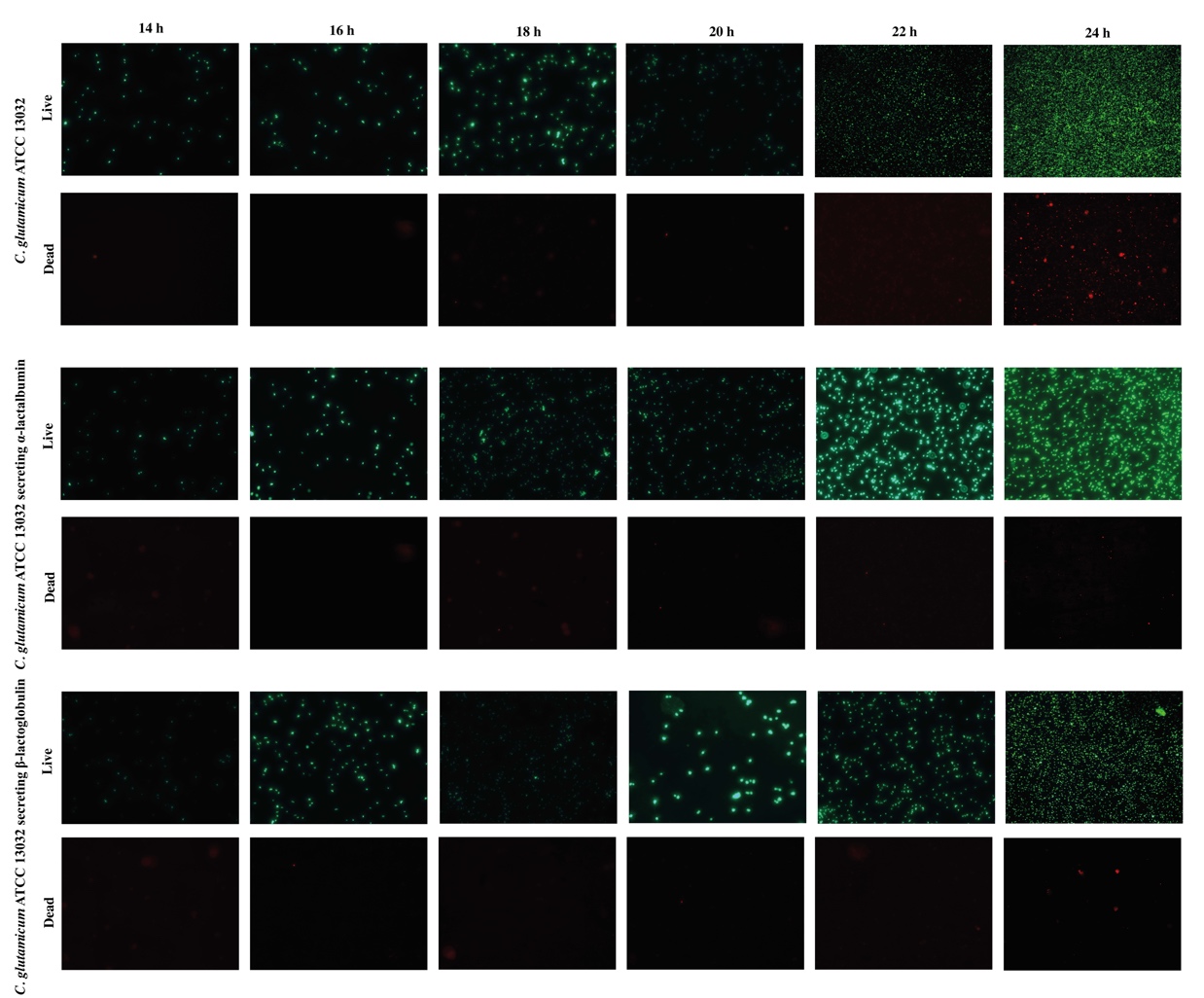


**Supplementary Figure 1.** Fluorescence microscopy of live/dead stained *C. glutamicum* ATCC 13032 (WT), WT secreting α-lactalbumin, and WT secreting β-lactoglobulin cells sampled every two hours in the exponential phase.


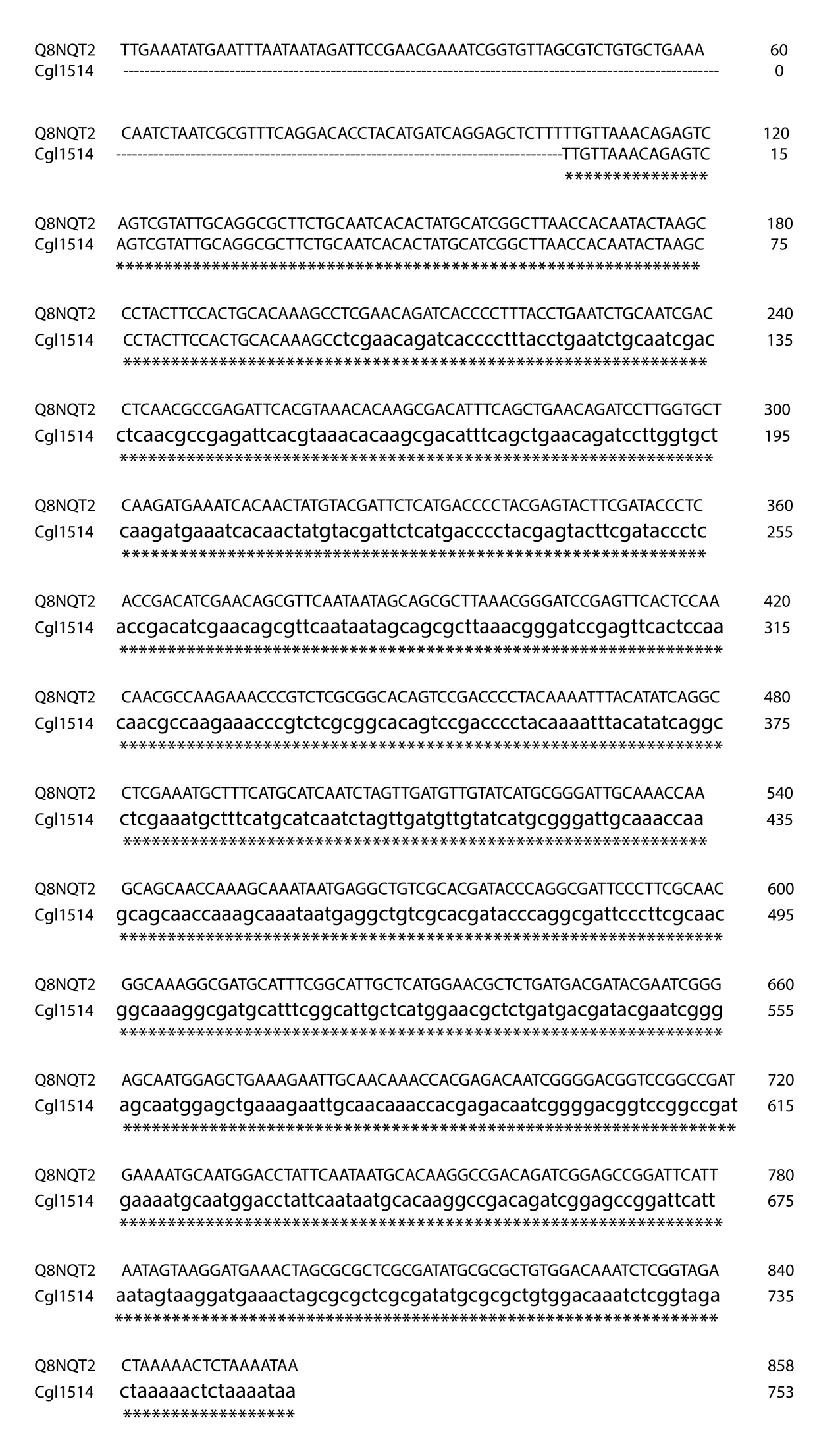


Supplementary figure 2 Sequence alignment of Cgl1514 and Q8NQT2 gene.

NCBI BLAST revealed an 87% match between the two genes. The 35 aa extensions at the N-terminal of Q8NQT2 includes genetic elements such as the SD sequence and the promoter of the gene, which inhibited the SP prediction in Signal P.
